# Association between gaseous air pollutants and idiopathic nephrotic syndrome in children: a 12-year population-based cohort study

**DOI:** 10.1186/s13052-022-01269-8

**Published:** 2022-05-12

**Authors:** Chieh Wang, Jeng-Dau Tsai, Lei Wan, Cheng-Li Lin, Chang-Ching Wei

**Affiliations:** 1 Department of Chinese Medicine, China Medical University Hospital, Taichung, Taiwan; 2 School of Medicine, Chung Shan Medical University, Taichung, Taiwan; 3 Department of Pediatrics, Chung Shan Medical University Hospital, Taichung, Taiwan; 4 School of Chinese Medicine, China Medical University, Taichung, Taiwan; 5 Management Office for Health Data, China Medical University Hospital, Taichung, Taiwan; 6 Institute of Biostatistics, China Medical University, Taichung, Taiwan; 7 Children’s Hospital, China Medical University Hospital, Taichung, Taiwan; 8 School of Medicine, China Medical University, No2, Yu-Der Road, Taichung, 40402 Taiwan

**Keywords:** Air pollution, Idiopathic nephrotic syndrome, Children, Cohort study, Sulfur dioxide, Total hydrocarbon, Methane

## Abstract

**Background:**

To date, there is insufficient knowledge about the association of air pollution and childhood nephrotic syndrome in the real world. This study aimed to evaluate the effects of the three common gaseous air pollutants, including sulfur dioxide, total hydrocarbon, and methane, on the risk of idiopathic nephrotic syndrome (INS) in children.

**Methods:**

We collected data from the Taiwan National Health Insurance Research Database and Taiwan Air Quality-Monitoring Database. Children younger than 18 years old, identified from January 1, 2000, were followed up until the first diagnosis of INS was established or until December 31, 2012. We measured the incidence rates and hazard ratios for INS stratified based on the quartiles (Q1–Q4) of air pollutant concentration. Multivariate Cox proportional hazards models were also applied by adjusting age, sex, monthly income, and urbanization.

**Results:**

Compared with participants exposed to Q1 concentrations, the adjusted hazard ratios (aHRs) for INS increased progressively along the four quartiles of sulfur dioxide, total hydrocarbon, and methane, from 1 (Q1) to 1.78 (Q4), 1 (Q1) to 3.49 (Q4), 1 (Q1) to 7.83 (Q4), respectively.

**Conclusions:**

Our study revealed that children with exposure to higher concentrations of sulfur dioxide, total hydrocarbon, and methane was associated with an increased risk of INS.

**Supplementary Information:**

The online version contains supplementary material available at 10.1186/s13052-022-01269-8.

## Introduction

Considering the rapid economic growth and urbanization process, air pollution has been noted as a major environmental risk to public health, accounting for 7 million premature deaths worldwide annually [1]. Exposure to air pollution is inevitable nowadays. Widespread air pollution may bring about both serious short-term and long-term health impacts on several organs and systems, including the diagnoses of several diseases such as chronic obstructive pulmonary disease, asthma, lung cancer, leukemia, immune system defects, and cardiovascular diseases [1]. Besides the well-known association between respiratory and cardiovascular diseases, an increasing body of evidence demonstrates that air pollution may be a risk factor for kidney diseases, such as acute kidney injury, chronic kidney disease (CKD), and kidney parenchyma cancer [2–6].

Nephrotic syndrome (NS) is the most common kidney disease in childhood. NS is generally divided into primary, idiopathic, secondary, and congenital NS [7]. Idiopathic NS (INS) is the most common form of NS observed in 90% of children, with an annual incidence of 2–16 new cases per 100,000 children [7]. Environmental factors, such as viral infection, allergic reactions, insect bites, vaccination, mercury exposure, and air pollution, have been reported as triggering factors of INS [7–10]. Identifying and avoiding potential environmental triggers is a crucial strategy to prevent the onset and development of INS.

In 2016, Xu et al. first proposed that long-term exposure to high levels of particulate matter (PM2.5) was associated with an increased risk of membrane nephropathy in China [8]. In 2018, Lin et al. reported that higher concentrations of PM2.5, nitric oxide (NO), nitrogen dioxide (NO2), and sulfur dioxide (SO2) are associated with an increased risk of NS in Taiwan [9]. However, little is known about air pollution and its impact on childhood INS, with the etiologies, management, and outcomes of INS significantly different between children and adults [7–11]. Minimal change disease (MCD) is a major cause of childhood INS, and greater than 90% of children with minimal change disease respond to corticosteroid therapy (steroid-sensitive NS) [7]. However, approximately 40% to 60% of steroid-sensitive NS presents a frequently relapsing or steroid-dependent clinical course, resulting in steroid burden and its adverse effects [7].

Despite recent advancements in understanding the ultrastructure of the filtration barrier, the precise etiology of INS remains unclear. Understanding the trigger factors causing INS is a prerequisite to prevent INS and develop targeted therapies. In this study, we aimed to select three representative groups of gaseous pollutants, including SO2 (non-volatile gas), total hydrocarbon (THC) (volatile gas), and methane (CH4) (greenhouse gas), and to determine the association between their exposures and the risk of INS in children. This nationwide cohort study used the Taiwan National Health Insurance Research Database (NHIRD) and the Taiwan Air Quality-Monitoring Database (TAQMD), a real-world dataset, to determine the long-term effects of gaseous air pollution on the incidence rates and risk of childhood INS.

## Methods and materials

### Data source

We conducted the retrospective cohort study by using the Children file, a representative data that comprises half of all children randomly selected from the year 2000 registry of beneficiaries of the Taiwan National Health Insurance Research Database (NHIRD). The NHID established in 1995 and covered more than 99% of the total population in Taiwan [12]. It contains all medical records, including de-identified demographic information (e.g., sex, birth dates, occupation and place of residence) and clinical information (e.g., diagnostic codes based on the international classification of disease, 9 th revision, clinical modification [ICD-9-CM], health management and treatment). For patient privacy protection, all the data in NHID was analyzed anonymously. This study was in accordance with the principles outlined in the Declaration of Helsinki and was approved by the China Medical University Hospital’s Institutional Review Board (CMUH104-REC2-115).

The TAQMD was released by the Taiwan Environmental Protection Administration, Executive Yuan. It includes daily concentrations of SO2, THC, and CH4 from 74 ambient air quality-monitoring stations distributed over Taiwan from 1998 to 2012. We averaged the daily air pollution data based on these recording stations. A residential area was defined based on the location of the clinic and hospital where a participant was treated acute nasopharyngitis (common cold) (ICD-9-CM code 460). Since acute nasopharyngitis is a common health problem, patients tend to visit the clinic and hospital close to where they live. A daily average air pollutant concentration was calculated by dividing the cumulative daily air pollutant concentration by the duration in 2000–2012. We linked two databases according to the residential areas of enrollees and the location of air quality-monitoring stations. Air pollutant concentrations were categorized into four groups based on quartiles. SO2 concentration was grouped into Q1 (< 3.38 ppb), Q2 (3.38–4.32 ppb), Q3 (4.32–6.03 ppb), and Q4 (> 6.03 ppb). THC concentration was grouped into Q1 (< 2.29 ppm), Q2 (2.29–2.38 ppm), Q3 (2.38–2.60 ppm), and Q4 (> 2.60 ppm). CH4 concentrations were grouped into Q1 (< 2.01 ppm), Q2 (2.01–2.06 ppm), Q3 (2.06–2.11 ppm), and Q4 (> 2.11 ppm).

### Study population, outcome of interest, endpoints, and confounding factors

**Fig. 1:**
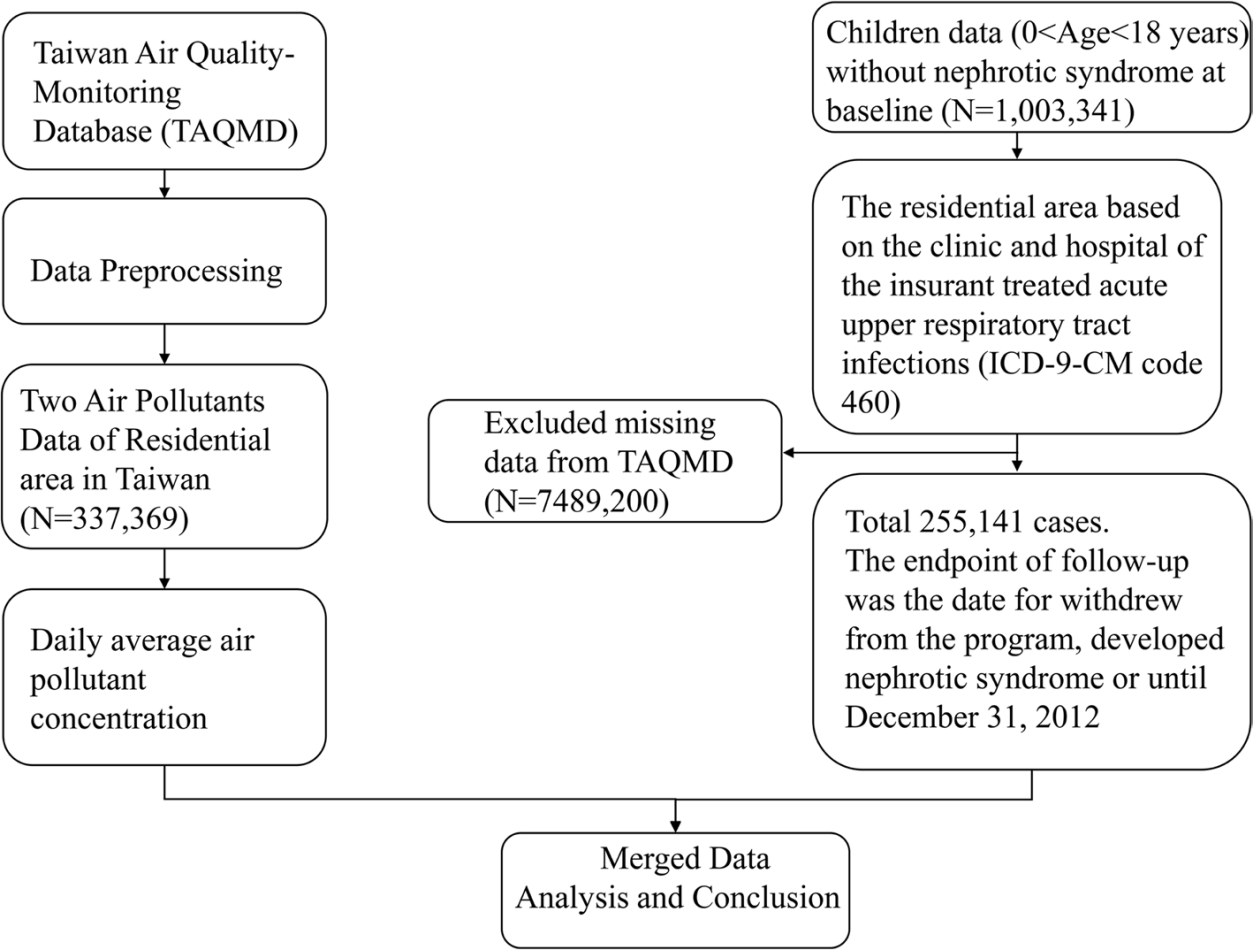
Flow diagram for study participants

In this study, we identified children younger than 18 years old on January 1, 2000 (baseline). All participants were followed from baseline until the diagnosis of INS made, withdrawal from the NHI, termination of insurance, death, or December 31, 2012. We also excluded children who had missing data, such as sex, address, and air pollution data, and individuals who had ever diagnosed INS before the baseline. INS was defined as ≥ 3 diagnoses of ICD-9-CM codes 581.3 (NS with lesion of minimal change glomerulonephritis) and/or 581.9 (NS with unspecified pathological lesion in kidney) in any diagnosis field during any inpatient or ambulatory claim process. The diagnosis of INS made under one-year-old was excluded as congenital NS. In any of the diagnostic fields with codes of 581.8 (secondary NS) was excluded as well. Therefore, the INS in our study was supposed to be MCD because MCD accounts for approximate 90% INS and mostly of MCD is recommended to start treatment without kidney biopsy. The flow diagram of current study was shown in Fig. 1.

The confounding factors mentioned in this study were sex, age, monthly income and urbanization level. Residential areas were also grouped into 4 levels of urbanization depending on the population density (people/km2), the ratio of elderly people, the ratio of people with different educational levels, the ratio of agricultural workers and the number of physicians per 100, 000 persons [13]. The highest degree of urbanization was level 1, and the lowest was level 4. Monthly income was also classified into 4 groups: < NT$14,400, NT$14,400–18,300, NT$18,301–21,000, and ≥ NT$21,000.

### Statistical analysis

The demographic data in our study included age, sex, urbanization level, monthly income, and daily average exposure to air pollutants. We performed χ2 testing in order to assess the differences in daily average concentration distributions for each air pollutant by quartile and urbanization. Moreover, we calculated the incidence density rate of INS (per 1000 person-years) according to each quartile of daily average concentrations of the three air pollutants. Cox proportional hazard regression models were also used to estimate the hazard ratios (HRs) and 95% confidence intervals (CIs) for INS in Q2–Q4 levels of air pollutant concentrations compared to the Q1 level. Univariable and Multivariable logistics regression was used to evaluate the effects of air pollutant concentration on the risk of INS, as indicated by the odds ratios (ORs) and 95% confidence intervals (CIs). All analyses were performed using SAS 9.3 (SAS Institute Inc, Cary, NC) and the Statistical Package for the Social Science (Version 15.1; SPSS Inc, Chicago, IL). All statistical tests were considered statistically significant when 2-tailed *p* values were < 0.05.

## Results

During the study period, 264 children (0.1%) were diagnosed with INS in a cohort of 255,141 children. Participants’ sociodemographic data reveal in Table 1. The mean age of the participants was 6.43 years old (standard deviation, 3.38). The mean participant follow-up period was 10.8 years (standard deviation, 2.78). There was slightly higher proportion of boys than girls (51.6% vs. 48.4%), which is similar to the national demographic data released by the Taiwan Ministry of Internal Affairs (the ratio of male to female under 15 is approximately 1.09:1). Besides, most participants lived in densely populated area with higher degree of urbanization level (Level 1 and Level 2, 65.3%).

**Table 1:** Baseline demographics and exposure of air pollutants among participants

***N* = 255,141**		***n***	**%**
Gender	Boys	131,696	51.6
Age, years	mean, SD^a^	6.43	3.38
3–6		133,612	52.4
7–12		103,217	40.5
> 12		18,312	7.18
Monthly income (NTD)^b^	< 14,999	213,695	83.8
	15,000 − 19,999	31,229	12.2
	≥ 20,000	10,217	4.00
Urbanization level	1 (highest)	84,887	33.3
	2	81,626	32.0
	3	48,375	19.0
	4 (lowest)	40,253	15.8
Exposure			
SO_2_ level (daily average)	mean, SD	5.59	2.42
THC level (daily average)	mean, SD	2.42	0.23
CH4 level (daily average)	mean, SD	2.03	0.13
Participant follow-up years	mean, SD	10.8	2.78
Outcome			
Nephrotic syndrome		264	0.10

^a^*SD* standard deviation, ^b^Monthly income: new Taiwan Dollar (NTD), 1 NTD is equal to 0.03 USD

Supplementary Tables 1, 2, and 3 demonstrate the baseline characteristics of participants exposed to 4 quartile levels of SO2, THC, and CH4 concentrations, respectively.

Table 2 shows the incidence rates and risk of INS among the four air pollutant concentrations. The incidence rate for INS increased with CH4 exposure concentration, from 0.32 (Q1) to 2.39 (Q4) per 100,000 person-years, respectively. Participants exposed to Q4 concentrations were associated with 1.78- and 7.83-fold higher risk of INS in SO2 (adjusted HR = 1.78, 95% CI = 1.20–2.64) and CH4 (adjusted HR = 7.83, 95% CI = 5.02–12.2), respectively, compared with those exposed to Q1 concentrations. In the THC group, relative to Q1 concentrations, the adjusted HRs were 2.09 (95% CI = 1.33–3.29), 3.61 (95% CI = 2.42–5.37), and 3.49 (95% CI = 2.24–5.42) for Q2, Q3, and Q4 levels, respectively. Then we stratified the 12-year follow-up period into three periods, each of 4 years. The results revealed increased IR and HR for INS were still associated with increased concentration levels of 3 gaseous pollutants during each 4-year observation period (Table 3). In order to estimate the overall ORs during the 12-year follow-up period, we used logistic regression models to detect the relationship between the concentration of 3 gaseous pollutants and INS (Table 4). The adjusted ORs in SO2, THC and CH4 group were 1.03 (95% CI = 0.99–1.07), 3.92 (95% CI = 2.28–6.72), and 999.9 (95% CI = 327.0–999.9), respectively.

**Table 2:** Differences in nephrotic syndrome incidences and associated HRs in participants exposed to various daily average concentrations of air pollutants in 2000–2012

Year 2000–2012	Event	PY^a^	IR^b^	cHR^c^	95%CI	aHR^d^	95% CI^e^
**SO**_**2**_							
Quartile 1, < 3.38 ppb	33	431,707	0.76	Reference group		Reference group	
Quartile 2, 3.38–4.32 ppb	39	589,450	0.66	0.87	(0.55, 1.38)	0.89	(0.56, 1.42)
Quartile 3, 4.32–6.03 ppb	83	914,846	0.91	1.19	(0.80, 1.78)	1.24	(0.82, 1.87)
Quartile 4, > 6.03 ppb	109	826,014	1.32	1.73	(1.17, 2.55)**	1.78	(1.20,2.64)**
**THC**							
Quartile 1, < 2.29 ppm	31	780,924	0.40	Reference group		Reference group	
Quartile 2, 2.29–2.38 ppm	48	583,193	0.82	2.08	(1.32, 3.26)**	2.09	(1.33, 3.29)**
Quartile 3, 2.38–2.60 ppm	121	889,830	1.36	3.47	(2.34, 5.16)***	3.61	(2.42,5.37)***
Quartile 4, > 2.60 ppm	64	508,071	1.26	3.25	(2.12, 5.00)***	3.49	(2.24,5.42)***
**CH4**							
Quartile 1, < 2.01 ppm	23	708,759	0.32	Reference group		Reference group	
Quartile 2, 2.01–2.06 ppm	30	765,925	0.39	1.20	(0.70, 2.07)	1.21	(0.70, 2.08)
Quartile 3, 2.06–2.11 ppm	77	726,349	1.06	3.29	(2.06, 5.24)***	3.33	(2.09,5.31)***
Quartile 4, > 2.11 ppm	134	560,985	2.39	7.68	(4.93, 12.0)***	7.83	(5.02,12.2)***

^a^*PY* person-years, ^b^*IR* incidence rate, (per 10,000 person-years), ^c^*cHR* crude hazard ratio, ^d^*aHR* adjusted hazard ratio, adjustment for age, sex, monthly income, and urbanization level, ^e^*CI* confidence interval; **p < 0.01; *** *p* < 0.001

**Table 3:** Differences in nephrotic syndrome incidences and associated HRs in participants exposed to various daily average concentrations of air pollutants stratified by 3 period of time in 2000–2012

	**Event**	**PY**^**a**^	**IR**^**b**^	**cHR**^**c**^	**95%CI**	**aHR**^**d**^	**95% CI**^**e**^
**Year 2000–2004**							
**SO**_**2**_							
Quartile 1, < 3.38 ppb	7	155,855	0.45	Reference group		Reference group	
Quartile 2, 3.38–4.32 ppb	12	211,515	0.47	1.26	(0.50, 3.21)	1.37	(0.54, 3.51)
Quartile 3, 4.32–6.03 ppb	24	338,017	0.71	1.58	(0.68, 3.67)	1.81	(0.77, 4.25)
Quartile 4, > 6.03 ppb	38	304,390	1.25	2.78	(1.24, 6.22)*	3.11	(1.37, 7.04)**
**THC**							
Quartile 1, < 2.29 ppm	3	267,526	0.11	Reference group		Reference group	
Quartile 2, 2.29–2.38 ppm	3	200,539	0.15	1.33	(0.27, 6.60)	1.37	(0.28, 6.76)
Quartile 3, 2.38–2.60 ppm	51	336,769	1.51	13.5	(4.21, 43.2)***	16.3	(5.05, 52.5)***
Quartile 4, > 2.60 ppm	24	204,943	1.17	10.4	(3.14, 34.6)***	14.6	(4.32, 49.4)***
**CH4**							
Quartile 1, < 2.01 ppm	0	245,282	0.00				
Quartile 2, 2.01–2.06 ppm	5	264,136	0.19	Reference group		Reference group	
Quartile 3, 2.06–2.11 ppm	19	261,444	0.73	3.84	(1.43, 10.3)**	4.07	(1.52, 10.9)**
Quartile 4, > 2.11 ppm	57	238,914	2.39	12.6	(5.04, 31.3)***	15.0	(5.96, 37.7)***
**Year 2005–2008**							
**SO2**							
Quartile 1, < 3.38 ppb	8	144,717	0.55	Reference group		Reference group	
Quartile 2, 3.38–4.32 ppb	8	201,468	0.40	0.72	(0.27, 1.91)	0.71	(0.27, 1.90)
Quartile 3, 4.32–6.03 ppb	26	307,104	0.85	1.53	(0.69, 3.39)	1.50	(0.67, 3.37)
Quartile 4, > 6.03 ppb	48	281,274	1.71	3.09	(1.46, 6.53)**	3.00	(1.41, 6.40)**
**THC**							
Quartile 1, < 2.29 ppm	6	262,547	0.23	Reference group		Reference group	
Quartile 2, 2.29–2.38 ppm	12	196,383	0.61	2.67	(1.00, 7.12)*	2.70	(1.01, 7.18)*
Quartile 3, 2.38–2.60 ppm	43	301,098	1.43	6.27	(2.67, 14.7)***	6.12	(2.59, 14.5)***
Quartile 4, > 2.60 ppm	29	174,535	1.66	7.31	(3.03, 17.6)***	7.02	(2.86, 17.2)***
**CH4**							
Quartile 1, < 2.01 ppm	5	241,793	0.21	Reference group		Reference group	
Quartile 2, 2.01–2.06 ppm	8	254,354	0.31	1.52	(0.50, 4.65)	1.52	(0.50, 4.65)
Quartile 3, 2.06–2.11 ppm	22	248,155	0.89	4.29	(1.62, 11.3)**	4.19	(1.58, 11.1)**
Quartile 4, > 2.11 ppm	55	190,262	2.89	14.1	(5.66, 35.3)***	13.4	(5.35, 33.7)***
**Year 2009–2012**							
**SO2**							
Quartile 1, < 3.38 ppb	18	131,135	1.37	Reference group		Reference group	
Quartile 2, 3.38–4.32 ppb	19	176,467	1.08	0.78	(0.41, 1.49)	0.79	(0.41, 1.51)
Quartile 3, 4.32–6.03 ppb	33	269,725	1.22	0.89	(0.50, 1.58)	0.90	(0.50, 1.62)
Quartile 4, > 6.03 ppb	23	240,350	0.96	0.69	(0.37, 1.29)	0.99	(0.88, 1.12)
**THC**							
Quartile 1, < 2.29 ppm	22	250,851	0.88	Reference group		Reference group	
Quartile 2, 2.29–2.38 ppm	33	186,270	1.77	2.02	(1.18, 3.47)*	2.02	(1.18, 3.47)*
Quartile 3, 2.38–2.60 ppm	27	251,963	1.07	1.22	(0.69, 2.14)	1.22	(0.69, 2.15)
Quartile 4, > 2.60 ppm	11	128,593	0.86	0.97	(0.47, 2.00)	0.97	(0.46, 2.02)
**CH4**							
Quartile 1, < 2.01 ppm	18	221,684	0.81	Reference group		Reference group	
Quartile 2, 2.01–2.06 ppm	17	247,435	0.69	0.85	(0.44, 1.65)	0.85	(0.44, 1.66)
Quartile 3, 2.06–2.11 ppm	36	216,749	1.66	2.04	(1.16, 3.59)*	2.07	(1.17, 3.65)*
Quartile 4, > 2.11 ppm	22	131,809	1.67	2.05	(1.10, 3.82)*	2.05	(1.10, 3.83)*

^a^*PY* person-years, ^b^*IR* incidence rate, (per 10,000 person-years), ^c^*cHR* crude hazard ratio, ^d^*aHR* adjusted hazard ratio, adjustment for age, sex, monthly income, and urbanization level, ^e^*CI* confidence interval. *p < 0.05; **p < 0.01; ***p < 0.001

**Table 4:** Comparisons of differences in of nephrotic syndrome incidences and associated ORs in participants exposed to various concentrations of air pollutants using a logistic regression

**Pollutant levels**	**cOR**^c^	**95%CI**^e^	**aOR**^d^	**95%CI**^e^
SO_2_	1.02	(0.98, 1.06)	1.03	(0.99, 1.07)
THC	2.79	(1.68, 4.63)***	3.92	(2.28, 6.72)***
CH4	537.9	(134.2, 999.9)***	999.9	(327.0, 999.9)***

^c^*cOR* crude odds ratio, ^d^*aOR* adjusted odds ratio, adjustment for age, sex, monthly income, and urbanization level, ^e^*CI* confidence interval. *p < 0.05; **p < 0.01;***p < 0.001

## Discussion

Air pollution has become one of the most significant global environmental hazards on human health [1]. Southeast Asia, the most polluted region in the world, recorded a total of 2.6 and 3.3 million deaths related to outdoor and indoor air pollution respectively in 2012 [1, 14]. Taiwan, an island country in East Asia, is geographically close to the most polluted area. The most important sources of air pollution are particulate pollution, gaseous pollution, and heavy metals [14]. In the current study, we selected three representative groups of gaseous pollution, including SO2 (non-volatile gas), THC (volatile gas), and CH4 (greenhouse gas), and investigated the impact of their exposure on the risk of INS in children. Our cohort study revealed that Taiwanese children exposed to higher concentrations of SO2, THC, and CH4 were at increased risk of developing INS, regardless if potential confounding factors such as age, sex, monthly income, and urbanization level were adjusted. Our results demonstrate that a clear dose–response relationship exists between concentration of air pollution and risk of INS.

When we looked at the differences in NS incidences and associated HRs in participants exposed to various daily average concentrations of air pollutants stratified by 3 period of time in 2000–2012, we noticed the HR for the onset of INS was not as significantly associated with the concentration of air pollution when the cohort following for 9–12 years (Year 2009–2012) as following for 1–4 years and 5–8 years (Year 2000–2004 and Year 2005–2008). The explanation was because age at initial presentation has an important impact on analysis of disease distribution frequency of epidemiological studies. MCD is a major cause (approximately 90%) of INS [7]. MCD mostly appears in children younger than 10 years, with the peak incidence of 2 to 6 years of age (approximately 70%) [7].

SO2 is generated from industrial sulfur-based products. SO2 is a well-known environmental pollutant that can be easily obtained from drinking water and inhalation. Several animal and epidemiological studies have demonstrated that SO2 causes not only respiratory system disease but also multiple organ damage in the brain, heart, kidney, liver, spleen, and testis [15–17]. SO2 may result in an imbalance of pro- and antioxidant systems, subsequently exerting its toxic effects through oxidative stress and inflammatory responses [18, 19]. Previous studies in mice showed that SO2 exposure induced serious ultrastructural lesions in renal proximal tubular lining cells, while glomeruli and distal tubular lining cells were damaged in a dose-dependent manner [15]. Moreover, SO2 can be immediately converted into HSO3-, SO32-, and H + in the interstitial fluid after entering the body, inducing cytosine deamination into uracil, further leading to deoxyribonucleic acid damage [20, 21].

THCs, which are often referred to as volatile organic compounds are a large group that have a significant impact on environmental and human health due to their toxic, mutagenic, and carcinogenic properties [22, 23]. Due to rapid industrialization and urbanization, total hydrocarbons (THCs) are recently responsible for the great majority of the global energy consumption (approximately 85%). Based on previous studies, hydrocarbons may have toxic effect on the glomerular basement membrane. It might give the explanation for that the cumulative incidence of THC increases more rapidly in the last 6 years. In addition, we also found that participants exposed to higher concentrations of THC had higher accumulative incidence of INS than those exposed to lower concentrations in our study. Based on the two case reports published in the late twentieth century, heavy occupational exposure to hydrocarbons was highly associated with NS [24, 25]. The mechanisms of hydrocarbon-induced glomerulonephritis are considered to be threefold, including antibody formation in alveolar cells and glomerular basement membrane due to chemical damage, glomerular deposition of immune complexes, and direct toxic effect of hydrocarbons on the glomerular basement membrane [26, 27].

CH4 is the second largest contributor to global warming and its concentration in the atmosphere has been increasing rapidly in the last decade [28]. CH4 emissions can be grouped into anthropogenic and natural sources. Anthropogenic activities, such as fossil fuel production, agriculture, wastewater production, and biomass burning, produce the majority of CH4 emissions, accounting for 50%–65% of the total CH4 [29]. Human activities in urban areas are recognized as a globally important source of CH4 to the atmosphere [29]. Although CH4 is generally considered nontoxic, little is known about the adverse health effects of direct CH4 exposure [30]. In our study, we first identified that CH4 exposure might lead to an increased risk of NS development. The following reasons might explain this finding. Firstly, infections are recognized most frequent triggers of NS relapse, particular viral upper respiratory tract infection. CH4 accelerates global warming and subsequent changes in climate, that may alter the incidence and severity of respiratory infections by affecting vectors and host immune responses [30, 31]. Children appear to be particularly vulnerable to rapid fluctuations in ambient temperature. For example, CH4 from rapid industrialization and urbanization gives rise to the epidemiology of infectious diseases, such as EBV, CMV and HHV7 infection [31, 32]. The higher potential for pathogen transmission probably would be a trigger for the onset or relapse of NS [7]. Secondly, NS can be precipitated by allergic reactions and children with NS also may show increased serum immunoglobulin E levels [7, 11]. Allergic disorders are common in children with NS, especially within the first year after diagnosis [11].The prevalence of asthma and allergies has increased during the past decades, particularly in developed countries. Global warming is linked to the emission of hydrocarbon combustion products, since both carbon dioxide and heat increase pollen emission into the atmosphere, and all these particles make up PM10 [28–30]. A rise in the concentrations of pollens and pollutants in the air parallels the increase in the number of people presenting with allergic symptoms, which results in increased risks of NS.

From the literature, only few studies have analyzed the effect of air pollution on the glomerulopathy in adults [8, 9]. Little is known about air pollution and its impact on childhood INS, with the etiologies, management, and outcomes of INS significantly different between children and adults [7–10]. Xu et al. found that long-term exposure to high levels of PM2.5 was associated with an increased risk of membranous nephropathy (MN), whereas the proportions of other major glomerulopathies remained stable regardless of the PM2.5 level [8]. From their adult series, MN was the leading cause of NS in adults aged > 40 years, while MCD was the most common histologic diagnosis among adults aged ≤ 39 years [8]. Exposure to the M-type phospholipase A2 receptor (PLA2R1) is critical for triggering the pathogenesis of PLA2R1-associated MN. Inflammation induced by air pollution, such as PM2.5, has been proposed to alter the microenvironment of PLA2R1-expressing cells [33]. PM2.5 also causes early kidney damage through oxidative stress or inflammation in the kidney microenvironment [34]. Lin et al. reported that higher concentrations of PM2.5, NO, NO2, and SO2 are associated with an increased risk of NS in adults [9]. However, in their study, the pathologic type of NS was not associated with air pollution [9]. In our study, we found that children exposed to higher concentrations of SO2, THC, and CH4 were associated with the risks of INS, mostly MCD. Although the exact biological mechanisms of air pollution and its effects on NS remain unclear, it is generally accepted that air pollutants entering the respiratory tract can be absorbed into the bloodstream through alveolar capillaries, resulting in systemic inflammation and oxidative stress [35–37]. An increase of reactive oxygen species (ROS) and the imbalance of ROS and ROS-inhibitory system were found in the blood of MCD patients [38, 39]. The systemic inflammation and oxidative stress disrupt the glomerular basement membrane and reduce de novo proteoglycan production, leading to the increase of glomerular basement membrane permeability and therefore cause proteinuria [40].

Overall, the importance and distinctiveness of our study were based on several aspects. First, our study appears to be the first population-based study assessing the association between air pollutants and INS in children. Children, one of the most susceptible subgroups of the population due to their immature respiratory, immune, reproductive, central nervous, and digestive systems, have a higher inhalation and resting metabolic rate of oxygen consumption per unit body weight than adults [41]. Second, except for nitrogen oxide, ozone, and particulate matter, which have been studies previously, our current study demonstrated the association of three types of gaseous pollutants, SO2, THC, and CH4, and INS. Third, in this study, we assessed the real-world data from the NHI program as our datasets; hence, the potential for selection bias could be minimized. Furthermore, we also tried to adjust for the confounding factors including sex, age, monthly income, and urbanization level to alleviate possible bias as well.

Our study had several limitations. First of all, the migration of our study population during the study period may be neglected. However, the bias could be minimized based on the 12-year compulsory education in Taiwan. The majority of children would stay studying in the fixed school district till the compulsory education ended. And the fact, change the place of residence is not quite common in children in Taiwan. Second, we were unable to acquire the individual results of kidney biopsy, which is often recommended in patients with NS to establish the pathologic subtype of the disease, to assess disease activity, or to confirm the diagnosis of diseases. Since kidney biopsy is usually not indicated for first presentation of childhood INS and empirical steroid treatment can be considered prior to kidney biopsy. Nevertheless, according to a previous study, even though most of children with INS had been coded as unspecified pathological lesion in kidney, their pathology should be MCD [11]. Third, several potential risk factors for NS, such as familial predisposition, genetic mutations, lifestyle, diet preference, previous over-the-counter medication, family history, exposure to other toxic substances, such as water pollution, heavy metals and smoking/tobacco exposure, and known viral and other infections like CMV, syphilis, malaria etc., could not be estimated in this study due to the insufficient information available in the NHIRD.

## Conclusions

In conclusion, this is the first study to demonstrate the association between INS and gaseous air pollutants in children. Our findings indicate that exposure to higher concentrations of SO2, THC, and CH4 might lead to an increased risk of INS development. These findings demonstrate some useful information for public health to monitor and further improve air quality. Further experimental studies are warranted to elucidate the underlying mechanisms and possibly identify the components in air pollution that are responsible for the pathogenesis of INS.

## Supplementary Information

**Additional file 1.**

## Data Availability

Not applicable.

## Abbreviations

CKD: Chronic kidney disease
CIs: Confidence intervals
HRs: Hazard ratios
INS: Idiopathic NS
CH4: Methane concentrations
MN: Membranous nephropathy
MCD: Minimal change disease
NS: Nephrotic syndrome
NO: Nitric oxide
NO2: Nitrogen dioxide
PM2.5: Particulate matter
ROS: Reactive oxygen species
PLA2R1: Phospholipase A2 receptor
SO2: Sulfur dioxide
THC: Total hydrocarbon
NHIRD: Taiwan National Health Insurance Research Database
TAQMD: Taiwan Air Quality-Monitoring Database
