# Supplementary material for: Association between gaseous air pollutants and idiopathic nephrotic syndrome in children: a 12-year population-based cohort study
Source: Ital J Pediatr. 2022 May 12;48:70. doi: 10.1186/s13052-022-01269-8 (PMC9097133; doi:10.1186/s13052-022-01269-8)
Supplement: Supplementary file 1 — Additional file 1. [file 13052_2022_1269_MOESM1_ESM.docx]

Supplementary Table 1. Baseline characteristics of participants exposed to various annual average concentrations of

sulfur oxides (SO_2_)

|  |  | SO_2_  N=255141 | | | | | | | | | |
| --- | --- | --- | --- | --- | --- | --- | --- | --- | --- | --- | --- |
|  |  | Q1 | | Q2 | | Q3 | | Q4 | | p-value |  |
|  | |  |  |  |  |  |  |  |  |  |  |
| Variable | | n | % | n | % | n | % | n | % |  |  |
| Age | mean, SD^a^ | 6.22 | 3.32 | 6.16 | 3.13 | 6.55 | 3.48 | 6.59 | 3.46 | <0.001 |  |
| Boys |  | 20360 | 51.8 | 27456 | 51.6 | 43898 | 51.5 | 39982 | 51.7 | 0.61 |  |
| Monthly income (NTD)^b^ | |  |  |  |  |  |  |  |  | <0.001 |  |
|  | < 14,999 | 33984 | 86.5 | 45629 | 85.7 | 70333 | 82.5 | 63749 | 82.5 |  |  |
|  | 15,000−19,999 | 4005 | 10.2 | 5836 | 11.0 | 11190 | 13.1 | 10198 | 13.2 |  |  |
|  | ≥ 20,000 | 1298 | 3.30 | 1762 | 3.31 | 3785 | 4.44 | 3372 | 4.36 |  |  |
| Urbanization level | |  |  |  |  |  |  |  |  | <0.001 |  |
|  | 1 (highest) | 8568 | 21.8 | 18741 | 35.2 | 34589 | 40.6 | 22989 | 29.7 |  |  |
|  | 2 | 10257 | 26.1 | 15065 | 28.3 | 25713 | 30.1 | 30591 | 39.6 |  |  |
|  | 3 | 6257 | 15.9 | 83339 | 15.7 | 17047 | 20.0 | 16732 | 21.6 |  |  |
|  | 4 (lowest) | 14205 | 36.2 | 11082 | 20.8 | 7959 | 9.33 | 7007 | 9.06 |  |  |
| Outcome |  |  |  |  |  |  |  |  |  |  |  |
| Nephrotic syndrome | | 33 | 0.08 | 39 | 0.07 | 83 | 0.10 | 109 | 0.14 | <0.001 |  |

Chi-square test; ^a^One-way ANOVA

^b^Monthly income: new Taiwan Dollar (NTD), 1 NTD is equal to 0.03 USD.

Supplementary Table 2. Baseline characteristics of participants exposed to various annual average concentrations of total hydrocarbons (THC)

|  |  | THC  N=255141 | | | | | | | | |
| --- | --- | --- | --- | --- | --- | --- | --- | --- | --- | --- |
|  |  | Q1 | | Q2 | | Q3 | | Q4 | | p-value |
| Variable | | n | % | n | % | n | % | n | % |  |
| Age | mean, SD^a^ | 5.51 | 2.60 | 5.54 | 2.73 | 6.88 | 3.62 | 7.73 | 3.84 | <0.001 |
| Boys |  | 34949 | 52.2 | 26073 | 51.8 | 44184 | 51.7 | 26490 | 50.5 | <0.001 |
| Monthly income (NTD)^b^ | |  |  |  |  |  |  |  |  | <0.001 |
|  | < 14,999 | 59656 | 89.1 | 44606 | 88.7 | 69546 | 81.4 | 39887 | 76.0 |  |
|  | 15,000−19,999 | 5950 | 8.88 | 4335 | 8.62 | 11825 | 13.9 | 9119 | 17.4 |  |
|  | ≥ 20,000 | 1366 | 2.04 | 1355 | 2.69 | 4038 | 4.73 | 3458 | 6.59 |  |
| Urbanization level | |  |  |  |  |  |  |  |  | <0.001 |
|  | 1 (highest) | 18871 | 28.2 | 11335 | 22.5 | 28510 | 33.4 | 26171 | 49.9 |  |
|  | 2 | 16331 | 24.4 | 19740 | 39.3 | 29892 | 35.0 | 15663 | 29.9 |  |
|  | 3 | 15454 | 23.1 | 7993 | 15.9 | 17673 | 20.7 | 7255 | 13.8 |  |
|  | 4 (lowest) | 16316 | 24.4 | 11228 | 22.3 | 9334 | 10.9 | 3375 | 6.43 |  |
| Outcome |  |  |  |  |  |  |  |  |  |  |
| Nephrotic syndrome | | 31 | 0.05 | 48 | 0.10 | 121 | 0.14 | 64 | 0.12 | <0.001 |

Chi-square test; ^a^One-way ANOVA

^b^Monthly income: new Taiwan Dollar (NTD), 1 NTD is equal to 0.03 USD.

Supplementary Table 3. Baseline characteristics of participants exposed to various annual average concentrations of methane (CH_4_)

|  |  | CH_4_  N=255141 | | | | | | | | |
| --- | --- | --- | --- | --- | --- | --- | --- | --- | --- | --- |
|  |  | Q1 | | Q2 | | Q3 | | Q4 | | p-value |
| Variable | | n | % | n | % | n | % | n | % |  |
| Age | mean, SD^a^ | 5.72 | 2.61 | 5.68 | 2.84 | 6.30 | 3.19 | 8.25 | 4.03 | <0.001 |
| Boys |  | 31948 | 52.1 | 34614 | 52.3 | 34069 | 51.6 | 31065 | 50.5 | <0.001 |
| Monthly income (NTD)^b^ | |  |  |  |  |  |  |  |  | <0.001 |
|  | < 14,999 | 53797 | 87.7 | 59054 | 89.2 | 55916 | 84.7 | 44928 | 73.0 |  |
|  | 15,000−19,999 | 6197 | 10.1 | 5333 | 8.05 | 7849 | 11.9 | 11850 | 19.3 |  |
|  | ≥ 20,000 | 1345 | 2.19 | 1846 | 2.79 | 2255 | 3.42 | 4771 | 7.75 |  |
| Urbanization level | |  |  |  |  |  |  |  |  | <0.001 |
|  | 1 (highest) | 18727 | 30.5 | 21093 | 31.9 | 24791 | 37.6 | 20276 | 32.9 |  |
|  | 2 | 15794 | 25.8 | 23667 | 35.7 | 22837 | 34.6 | 19328 | 31.4 |  |
|  | 3 | 15133 | 24.7 | 12058 | 18.2 | 10713 | 16.2 | 10471 | 17.0 |  |
|  | 4 (lowest) | 11685 | 19.1 | 9415 | 14.2 | 7679 | 11.6 | 11474 | 18.6 |  |
| Outcome |  |  |  |  |  |  |  |  |  |  |
| Nephrotic syndrome | | 23 | 0.04 | 30 | 0.05 | 77 | 0.12 | 134 | 0.22 | <0.001 |

Chi-square test; ^a^One-way ANOVA

^b^Monthly income: new Taiwan Dollar (NTD), 1 NTD is equal to 0.03 USD.
